# Amino Acid Patterns in Children with Autistic Spectrum Disorder: A Preliminary Biochemical Evaluation

**DOI:** 10.3390/nu17020274

**Published:** 2025-01-13

**Authors:** Simona Ferraro, Laura Saielli, Davide Biganzoli, Martina Tosi, Laura Guidi, Roberto Longo, Francesca Severino, Stephana Carelli, Maura Rossi, Livia Pisciotta, Emilia Ricci, Francesca Brustia, Elvira Verduci, Gianvincenzo Zuccotti, Michele Mussap, Cristina Cereda

**Affiliations:** 1 Department of Pediatrics, Buzzi Children’s Hospital, 20154 Milan, Italycristina.cereda@asst-fbf-sacco.it (C.C.); 2 Center of Functional Genomics and Rare Diseases, Buzzi Children’s Hospital, 20154 Milan, Italy; 3 Department of Health Sciences, University of Milan, 20146 Milan, Italy; 4 Corporate Information Systems, Buzzi Children’s Hospital, 20154 Milan, Italy; 5 Department of Biomedical and Clinical Sciences, University of Milan, 20157 Milan, Italy; francesca.severino@unimi.it; 6 Pediatric Clinical Research Center “Romeo ed Enrica Invernizzi”, University of Milan, 20157 Milan, Italy; 7 Child and Adolescent Neuropsychiatry Unit, ASST Fatebenefratelli Sacco, 20157 Milan, Italylivia.pisciotta@asst-fbf-sacco.it (L.P.); 8 Child Neuropsychiatry Unit, Epilepsy Center, San Paolo Hospital, Department of Health Sciences, University of Milan, 20142 Milan, Italy; emilia.ricci@asst-santipaolocarlo.it; 9 Child Neuropsychiatry Unit, University Hospital Maggiore della Carità, 28100 Novara, Italy; 10 Metabolic Diseases Unit, Buzzi Children’s Hospital, 20154 Milan, Italy; 11 Laboratory Medicine, Hospital Foundation Villa Salus, 30174 Venice, Italy; mumike153@gmail.com; 12 Molecular Unit, Department of Surgical Sciences, University of Cagliari, 09124 Cagliari, Italy

**Keywords:** amino acid profile, metabolism, autism spectrum disorder, neurological impairment, nutritional dysregulation

## Abstract

Background: The metabolism of plasma amino acid (AA) in children with autism spectrum disorder (ASD) has been extensively investigated, yielding inconclusive results. This study aims to characterize the metabolic alterations in AA profiles among early-diagnosed children with ASD and compare the findings with those from non-ASD children. Methods: We analyzed plasma AA profiles, measured by ion exchange chromatography, from 1242 ASD children (median age = 4 years; 81% male). Additionally, we studied AA profiles from 488 children, matched for age and free of ASD (control group). Principal component and cluster analysis were employed to explore potential associations within the ASD group and to identify subgroups. Results: We observed lower plasma levels of glutamine in children with ASD compared to non-ASD children (*p* < 0.001). Six essential, two conditionally essential, and four non-essential AA were found to be increased in children with ASD. The clustering analysis revealed two groups, labeled Neurological (NEU) and Nutritional (NUT), which included a majority of ASD children (94% and 78%, respectively). The NEU group exhibited high levels of taurine, aspartate, glutamic acid, and ornithine, while the NUT group showed elevated levels of branched-chain AA. Conclusions: In children with ASD, we identified some heterogeneous AA patterns that may serve as biochemical signatures of neurological impairment in some individuals, while in others they may indicate nutritional dysregulation.

## 1. Introduction

Autism Spectrum disorder (ASD) is a pervasive and heterogeneous neurodevelopmental disorder that affects individuals differently across a broad spectrum. The social cost associated with ASD is significant, impacting affected persons, their families, and the healthcare system [1]. The estimated prevalence of ASD is approximately 1.85% among children aged 8 years, with a male to female ratio of 4:1 [1]; these estimates, however, vary considerably worldwide [1]. In addition to the two core domains of deficits that make up the ASD diagnostic criteria—limited social and communication abilities and restricted or repetitive patterns of behavior and interests [1]—individuals with ASD often have co-occurring psychiatric or neurological conditions including epilepsy, attention-deficit/hyperactivity disorder (ADHD), anxiety, and depression [1]. Furthermore, approximately 49% of autistic individuals experience various gastrointestinal disorders, such as chronic constipation, chronic diarrhea, gastroesophageal reflux, nausea and/or vomiting, flatulence, chronic bloating, abdominal discomfort, ulcers, colitis, inflammatory bowel disease, and food intolerances [1]. The presence of these comorbidities can exacerbate the severity of ASD and contribute to its overall morbidity and heterogeneity [1].

In the past, a significant number of children with undetected inborn errors of metabolism (IEM) related to amino acid (AA) metabolism, including aminoacidopathies (e.g., phenylketonuria), could not receive a correct diagnosis of their underlying etiology due to similar neuro-behavioral presentations. According to current guidelines, amino acids (AA) profiling and the clinical evidence of pathologic levels of specific AA can support the differential diagnosis between ASD and aminoacidopathies (e.g., phenylketonuria) and guide nutritional therapeutic interventions [2,3,4,5].

In the absence of the pathologic AA levels associated with IEMs, the metabolism of plasma AA in children diagnosed with ASD has been widely investigated [5,6,7,8,9,10,11,12,13]. Unfortunately, the literature on AA metabolism in children and adults with ASD remains inconclusive and contradictory [9]. Discrepancies between studies are primarily due to the high heterogeneity of the individuals enrolled. In addition to the severity of the disorder, various factors contribute to the heterogeneous findings in AA profiles in ASD, including comorbidities, food selectivity, dietary supplementation, patient age, and study design (e.g., sample size, enrollment criteria, blood sample collection after overnight fasting, and statistical modeling) [10]. Another source of variability in ASD is the dysregulation of LAT protein complexes, which disrupts the transport of large neutral amino acids, including aromatic AA such as tryptophan (Trp), phenylalanine (Phe), and tyrosine (Tyr), as well as branched-chain amino acids (BCAA), across the blood–brain barrier [12]. Rare genetic mutations, such as those affecting the BCKDK gene, can also lead to changes in plasma AA levels, particularly BCAA, in individuals with autism [13].

The aim of this pilot study is to characterize potential metabolic alterations in the amino acid (AA) profiles of very young children diagnosed with autism spectrum disorder (ASD), compared to non-ASD children. To achieve this, we aim to compare the AA profiles detected in plasma samples collected from a sizable group of children diagnosed with ASD at an early stage with those from a control group of non-ASD children of the same age.

Preliminary biochemical evidence of altered AA patterns in a large case series of ASD could pave the way for further clinical studies investigating ASD phenotypes and nutritional approaches, leveraging insight from metabolic profiling [14].

## 2. Materials and Methods

### 2.1. Patients and Control Group

This study retrospectively analyzed data from AA determinations conducted at the Center of Functional Genomics and Rare Diseases Buzzi Children’s Hospital, Milan, Italy, from January 2016 to January 2023. Data were collected from 1242 children diagnosed with ASD across five specialistic neurological centers in Lombardy region, Italy. Following initial diagnosis, a plasma AA profile determination was recommended to identify any alterations potentially attributable to concomitant IEM of AA (i.e., phenylketonuria, tyrosinemia, and homocystinuria) [3,4].

ASD diagnosis was made according to the Diagnostic and Statistical Manual of Mental Disorders, 5th ed. (DSM-5) criteria and the Autism Diagnostic Observation Schedule-Second Version (ADOS-2) [15]. Children were excluded from the study if ASD was found to be associated with IEMs (i.e., phenylketonuria, homocystinuria, S-adenosylhomocysteine hydrolase deficiency, branched-chain a-keto acid dehydrogenase kinase deficiency, urea cycle disorders, and organic acidurias). The median age of ASD group was 4 years old (interquartile range (IQR) 3–6 y.o.), with 81% being male. The control group consisted of 488 children, free of ASD; whose plasma AA profiles were identified to be normal in the laboratory report. This report also included all the clinical information used to select the control group. The normal AA profile was determined by comparing the concentration of each AA with age-specific reference ranges determined for each AA [16]. Accordingly, the control group exhibited a wide distribution of normal concentrations for each AA, consistent with a typical profile. The median age of the control group was 3 years old (IQR 1–7 y.o.), with 63% being male.

### 2.2. Ethical Statement

This study was approved by the Local Ethics Committee LOMBARDIA 1 (protocol n #CET 5-2023 of 12 July 2023). The research was conducted using data collected during the daily clinical care of patients with ASD. All procedures adhered to the guidelines of the Local Ethical Committee and have been performed in accordance with the ethical standards as laid down in the 1964 Helsinki Declaration and its later amendments. Informed consent was obtained for the treatment of anonymized data from a parent or a legal guardian. Anonymization procedures were validated and approved. The study protocol was accepted as part of a larger clinical study on autism spectrum disorder.

### 2.3. AA Profiles in Plasma

Venous blood samples were collected into Li-heparin coated tubes from the cubital vein in the morning, following overnight fasting, at the neurologic clinical center where children diagnosed with ASD were admitted. Within 30 min of collection, the samples were centrifuged at 3500 rpm for 15 min to obtain plasma for subsequent analysis. The plasma was then sent to our laboratory and stored at 4–8 °C or frozen at −20 °C. Hemolyzed specimens were discarded.

Plasma was deproteinized prior to analysis by mixing 10% sulfosalicylic acid solution containing the required concentration of the internal standard (2-Amino-2-Deoxy-d-Gluconic Acid (SIGMA Aldrich, Merck Spa, Milan, Italy). The sample was mixed and incubated for 20 min at 4 °C to allow precipitate formation. After centrifuging at 3500 rpm for 15 min at 4 °C, the supernatant was filtered through a 0.2-micron membrane filter to remove any remaining particulate matter. Plasma AA levels were measured by using Biochrom 30PLUS AA amino acid ion exchange chromatography (IEC) [4] (Biochrom Ltd., Cambridge, UK; https://www.biochrom.co.uk, accessed on 17 May 2024).

Following the injection of 50 µL of sample in the ion-exchange chromatography system, the AA were separated according to the standard manufacturer protocol using changes in ionic strength, pH of the mobile phase, and the temperature of the lithium cation exchange column with column guard. After the column separation, the samples were derivatized with ninhydrin and the resulting colored complex was detected spectrophotometrically at 570 nm for all AA, except for proline and hydroxyproline, which were detected at 440 nm. The intensity of the color complex was proportional to the concentration of the AA in the sample. Identification and quantification of AA were based on the comparison of the retention times and the signal responses in the standard solutions with known AA concentrations, using single point calibration. The area under the peak of each individual AA was directly proportional to its concentration.

The method has a linearity range of 9.8 pmoles–10 nmoles and a limit of quantitation of 15 pmol. System calibration was performed using Physiological Amino acids Standards basics, acid and neutrals (SIGMA Aldrich, Merck Spa, Milan, Italy).

Quality control measures were implemented using certified reference materials, specifically amino acids ERNDIM IQCS^®^ (ERNDIM MCA Laboratory, Beatrixpark, BN, Winterswijk, The Netherlands) Plasma Control, lyophile, Levels I and II for human plasma amino acids. Only analytical runs with imprecision and inaccuracy <15% were accepted.

The values obtained for all AA fell within the certified control range. The plasma amino acids measured included: alanine (Ala), arginine (Arg), asparagine (Asn), aspartate (Asp), citrulline (Cit), glutamine (Gln), glutamic acid (Glu), glycine (Gly), histidine (His), isoleucine (Ile), leucine (Leu), lysine (Lys), methionine (Met), ornithine (Orn), phenylalanine (Phe), proline (Pro), serine (Ser), taurine (Tau), threonine (Thr), and valine (Val).

### 2.4. Statistical Methods

Univariate statistical analyses were performed to compare AA concentrations in children with ASD vs. the control group. Principal component analysis (PCA) was employed to explore potential relationships among the AA profiles of children within the ASD group. This method allowed us to reduce the dimensionality of the dataset by projecting each data point onto the first principal components (up to three) while preserving as much variation as possible. We explored the AA profiles of ADS patients by passively projecting them onto the spaces defined by the first three principal components (PCs). Additionally, hierarchical cluster analysis using Ward’s method was performed to generate a dendrogram, facilitating the estimation of the number of clusters within the sample. We made cuts at the points of change between successive fusion levels to define likely cluster boundaries. The resulting number of clusters was further sub-defined using k-means cluster analysis. The k-means algorithm was run 50 times with random starting points to achieve repeatability and stability in each model. Clusters defined by k-means algorithm were subsequently projected into subspaces defined by PC1, PC2, and PC3, calculated through PCA performed on ASD children. Further univariate statistical analysis was conducted to examine the different characteristics of the clusters identified in the study. A summary table was created, presenting the median and IQR for all AA and ages, stratified by cluster. Non-parametric Wilcoxon rank sum test and Bonferroni correction for multiple testing have been used to compare AA levels between 2 or more groups, respectively. Significant median differences between 2 or more groups were reported in terms of *p*-value and q-value, respectively.

K-means clustering was used to detect and characterize homogeneous groups based on overall AA profiles [17,18]. The number of clusters has been defined using an “elbow” method. All the statistical analyses were performed using R software (version 4.1.2, https://cran.r-project.org/, accessed on 15 April 2024). The packages used for multivariate analysis were ‘FactoMineR’ (version 2.4), ‘factoextra’ (version 1.0.7), and ‘stats’ (version 3.6.2).

## 3. Results

### 3.1. Univariate and Correlation Analysis

A total number of 1730 children were included in this study: 1242 children with a diagnosis of ASD and 488 children with a normal AA profile, free of ASD, who were considered the control group.

Only Gln levels were significantly lower in ASD children compared with non-ASD children (*p* < 0.001); Arg was found to be marginally lower in ASD children (*p* = 0.06). By contrast, six essential amino acids, two conditionally essential amino acids, and four non-essential amino acids were significantly elevated in the ASD group (see Table 1). Finally, plasma concentrations of Lys, Met, Thr, Arg, Gly, Pro, and Asn did not significantly differ between the two groups. Notably, plasma levels of Met, Pro, and Asn were closely comparable between the two groups. The correlation matrix performed for the overall set is provided in Supplementary Figure S1.

### 3.2. Multivariate Analysis—Principal Component Analysis

#### Autism Spectrum

The first three principal components (PCs) collectively accounted for 54.3% of the variance in the data (PC1 = 35.1%, PC2 = 10.8%, and PC3 = 10.3%) (see Supplementary Figure S2). Thr, Ser, BCAA, Glu, Lys, and Ala were identified as the primary variables contributing to the differences in the first three PCs, followed by secondary contributions from Met, Tyr, and Orn (refer to Supplementary Figure S3).

**Figure 1:**
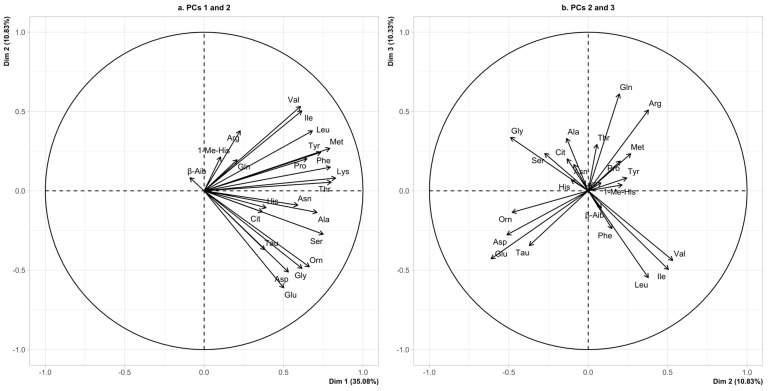
In these panels, the correlations are represented as vectors, with each variable projected onto subspaces defined by the principal components derived from the multivariate analysis (PCA). When variables are positively correlated, their vectors align in the same direction and on the same line (vectors grouped together). In contrast, negative correlated variables are positioned in the opposite direction (vectors positioned on opposite sides of the plot and in opposite quadrant). Uncorrelated variables tend to project perpendicularly, forming a 90° angle. The distance from the origin to each variable’s vector indicates its contribution to the principal component, with longer vectors representing a higher contribution from that variable. Abbreviations: “P-Ser”, Phosphoserine; “Tau”, #Taurine; “P-Etn”, #Phosphoethanolamine; “Asp”, #Aspartic Acid; “Thr”, #Threonine; “Ser”, #Serine; “Asn”, #Asparagine; “Glu”, #Glutamic Acid; “Gln”, #Glutamine; “Sar”, #Sarcosine; “Aad”, # alpha-Aminoadipic Acid; “Hyp”, #Hydroxyproline; “Pro”, #Proline; “Gly”, #Glycine; “Ala”, #Alanine; “Cit”, #Citrulline; “Abu”, #alpha-Aminobutyric Acid; “Val”, #Valine; “Cys-Cys”, #Cystine; “Met”, #Methionine; “Cysta”, #Cystathionine; “Ile”, #Isoleucine; “Leu”, #Leucine; “Tyr”, #Tyrosine; “β-Ala”, #beta-Alanine; “Phe”, #Phenylalanine; “β-Aib”, #beta-Aminoisobutyric Acid; “γ-Aib”, #gamma-Aminoisobutyric Acid; “Orn”, #Ornithine; “Lys”, #Lysine; “His”, #Histidine; “1-Me-His”, #1-Methylhistidine; “3-Me-His”, #3-Methylhistidine; “Car”, #Carnosine; “Arg”, #Arginine; and “Hcy” #Homocystine.

**Figure 2:**
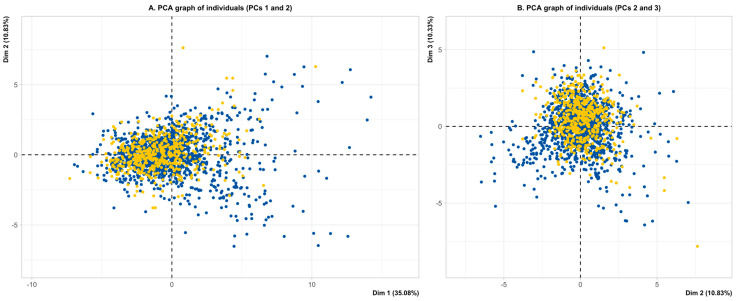
(**A**,**B**). Bidimensional representation of children with ASD (blue) along with a passive projection of the non-ASD group (yellow), based on the complete set of AA.

The projections of the original variables onto the three planes are shown in Figure 1a,b. In this projection, the first principal component (PC1) primarily reflected correlations between Thr, Lys, Ser, and Ala. PC2 was mainly characterized by correlations between Glu, Asp, Tau, and Orn, with an opposing relationship to Arg and, to some extent, BCAA. PC3 exhibited BCAA in opposition with Gln. Notably, BCAA displayed a nearly orthogonal (uncorrelated) pattern with respect to Tau, Asp, Glu, and Orn in this projection. Figure 2A,B show the distribution of ASD cases and passively projected subjects from the non-ASD group onto the three planes. Two elongated cloud profiles are evident in the right top and bottom corners. These profiles correspond to the highest BCAA levels (right top) and the highest levels of Tau, Asp, Glu, Gly, and Orn (right bottom). The same two profiles are seen in Figure 2B, where subjects are projected in the plane defined by the second and third PCs, with the highest BCAA (right bottom) and the highest Tau, Asp, Glu, and Orn (left bottom). Gly exhibits a separate behavior in this second plane. A shrinkage towards the axes’ centroid is evident for non-ASD children, as they were not included in the active projection. To further corroborate this observation, an additional analysis was performed by including subjects from the non-ASD group along with the active cases (Supplementary Figures S4–S6). This analysis confirms two elongated cloud profiles: one mainly represented by the BCAA (right top, Supplementary Figure S6a) and the other by Tau, Asp, Glu, and Orn (right bottom, Supplementary Figure S6a), both clearly illustrated by the selected cases.

### 3.3. K-Means Clustering

**Figure 3:**
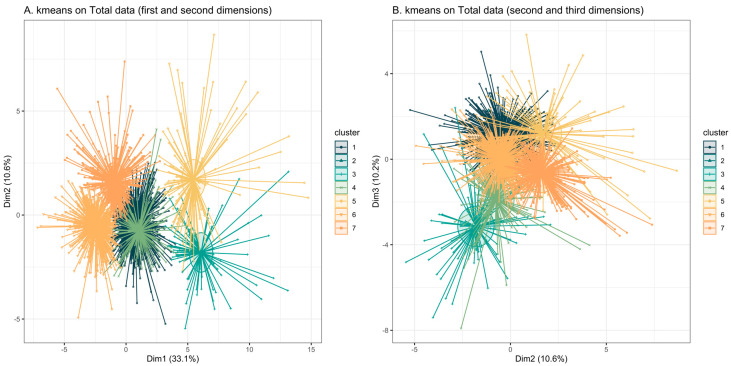
(**A**,**B**). Clustering results based on both ASD and non-ASD groups. Individuals are represented on the plot using principal components, with ellipses drawn according to Euclidean distances. Each individual is connected to their respective centroid.

Following the graphical analysis from the PCA plots, we performed a cluster analysis on overall case series, including all subjects from both the ASD and the non-ASD groups. This analysis allowed us to characterize subgroups based solely on the AA profile. First, we optimized the number of clusters using the heuristic elbow method, which suggested the presence of seven clusters. Then, based on this segmentation, we identified two distinct groups. The first one, marked by high plasma levels of Tau, Asp, Glu, and Orn, was called the Neurological (NEU) group, consisting of 103 subjects, 94% of whom were diagnosed with ASD (cluster 3, Figure 3A,B). NEU refers to the fact that most of these AA are precursors or involved in the synthesis and/or modulation of neurotransmitters. The second group was characterized by marked levels of Leu and Val (BCAA), which are essential AA derived from diet, and was called the Nutritional (NUT) group, including 78 subjects (cluster 4, Figure 3A,B), 78% of whom had ASD.

Notably, upon visual inspection, comparing Figure 3B (clusters) and Figure 1b (projections of the AA), we observed that the AA pattern of the NEU group was primarily characterized by Tau, Asp, Glu, and Orn while the AA pattern of NUT group was marked by Leu and Val (BCAA).

A comparison of plasma AA levels between the NEU and the NUT groups, as well as with the non-ASD children, is summarized in Table 2 and Table 3. When compared with the non-ASD group, the NEU group exhibited increased plasma levels of nearly all AA and a decrease in two conditionally essential amino acids, namely Arg and Gln (Table 2). More specifically, in NEU vs. non-ASD, the non-essential amino acids Asp and Glu increased by 3.4 and 3.3 times, respectively, and the conditionally essential AA Orn and Tau by 2.4 and 2.2 times (Table 3), respectively.

Based on the magnitude of (positive and negative) changes in AA plasma levels within the NEU subgroup when compared to the ASD and non-ASD groups (Table 3), we identified five AA (Arg, Glu, Orn, Asp, and Tau) as fingerprints of the NEU subgroup, which mainly includes ASD children (see Table 3). On the other hand, eight amino acids (Lys, Met, Thr, Ile, Leu, Val, Pro, and Ala) were identified as fingerprints of the NUT subgroup, representing ASD children with potential metabolic and nutritional dysregulation (see Table 3).

## 4. Discussion

The metabolism of AA is significantly disrupted in children with ASD; however, multiple factors, such as comorbidities, severity of core symptoms, and therapeutic treatments, may contribute to the variability observed in the literature. As a result, conclusive data are currently lacking. In this study, we enrolled a large cohort of children with ASD and compared their AA profiles with those of children affected by neurological and metabolic disorders (non-ASD group), aiming to identify specific metabolic alterations associated with ASD. Additionally, our cluster analysis enabled the characterization of distinct AA profiles in two subsets: NUT and NEU groups. These groups predominantly included ASD cases (78% and 94%, respectively), independent of other clinical factors. Our systematic approach contributes new insight to the existing literature, which typically compares ASD children with healthy controls. Such studies often provide limited information or can lead to misleading conclusions regarding various AA. For example, this is evident in the case of Gly, which plays a key role in brain development and regulation through multiple biochemical pathways [19]. In the early developmental stage, Gly acts as an excitatory neurotransmitter, transitioning to an inhibitory function as the nervous system matures. Gly also modulates glutamatergic neurotransmission as a co-agonist of N-methyl-D-aspartate (NMDA) receptors, and exerts anti-inflammatory, cytoprotective, antioxidant (as a precursor of glutathione), and immune-modulating effects [19]. Gly also contributes to the synthesis of 5-aminolevulinic acid, a precursor of heme and vitamin B12, in one-carbon metabolism and purine synthesis.

In our study, plasma levels of Gly did not exhibit significant differences between ASD and non-ASD groups, which is consistent with previous evidence [20]. However, in the NEU subgroup, Gly plasma levels were significantly higher than those found in the non-ASD children (31%), in the whole ASD group (30%), and in the NUT (34%) groups. Some authors have recently hypothesized that, in ASD patients, altered Gly concentrations may disrupt the excitation/inhibition balance in the brain [21]. 

In our preliminary study, Arg plasma levels did not display significant differences between the entire ASD and non-ASD groups, which is consistent with most of the published findings [7,20,22,23]. Only one study reported an 18% decrease in Arg levels in autistic children compared to neurotypical (NT) children [24], while two other investigations showed increased levels in ASD versus NT [25,26]. In our study, the NEU subgroup exhibited lower Arg levels compared to those in the non-ASD group (97% decrease), with an 87% decrease observed in the entire ASD group (Table 3). Our data did not identify Arg as a fingerprint of the NUT subgroup.

Arg serves as the precursor of several molecules, including urea, creatinine, and proteins, along with cell-signaling molecules such as Glu, agmatine, polyamines, and nitric oxide (NO), which act as the primary brain vasodilator [27]. It plays a crucial role in ammonia detoxification by converting into urea via the enzyme arginase and is involved in the regulation of cerebral blood flow. Specifically, NO, a potent vasodilator, is synthesized from Arg and plays a key role in maintaining blood circulation in the brain. High plasma levels of nitrite in autistic children have been linked to inducible NO production in response to intestinal infection, resulting in Arg depletion, the substrate of endothelial and neuronal NO production [28]. Thus, it is not surprising that Arg depletion, observed in the NEU group, may lead to alterations in the survival and functionality of neuronal cells.

ASD children showed a significant increase in Glu (5.6%) along with a significant decrease in Gln (7.6%) compared to non-ASD children. More importantly, our study suggests that the increase in Glu levels might be a specific metabolic signature in ASD, since a far higher increase in the NEU subgroup versus the whole ASD, non-ASD and NUT groups was observed (Table 3). On the other hand, this increase was comparatively modest in the NUT subgroup when compared to overall ASD and non-ASD children.

Glu is the brain’s primary excitatory neurotransmitter, and its homeostasis is crucial for optimal CNS functioning. Glu and Gln undergo recycling between neurons and astrocytes through the so-called Glu–Gln cycle, which links Glu’s cellular homeostasis to energy metabolism [29]. In this cycle, Glu is converted into Gln by the Gln synthetase expressed in astrocytes, whereas Gln is converted back into Glu by an enzyme predominantly located in the neurons [29]. Abnormally elevated concentrations of Glu can lead to the overstimulation of its receptors, resulting in increased levels of sodium and calcium ions, which form the basis for Glu excitotoxicity effects, ultimately causing neuronal damage and cell death [30,31]. It has been suggested that abnormal glutamatergic neurotransmission is implicated in the pathophysiology of ASD, and blood Glu levels might be a potential biomarker of ASD [32]. Early studies reported a significant increase in Glu blood levels in children with ASD compared to controls, together with reduced levels of Gln [6]. Further studies have confirmed this trend [8,33], showing both the increase in the Glu/Gln ratio and a close correlation between higher Glu plasma levels and the severity of ASD [2]. Consistent with our findings, revealing a more pronounced decrease in Gln levels in the NEU group versus other groups, the depletion (even marginal) of Gln in children and adults with ASD has been frequently documented in the literature [6,7,33]. Gln is the most abundant AA in the human body, being correlated to multiple functions and molecular targets in the metabolic pathways [34]. Gln is converted into Orn, which serves as the precursor of Arg [34]. In the brain, Gln prevents the accumulation of Glu, and, thus, Gln concentration may be considered a reliable indicator of synaptic glutamatergic neurotransmission. Alterations in astrocyte Gln levels can lead to mitochondrial dysfunction, with loss of energy production, stress response, and, ultimately, neuronal death. Gln depletion reduces the synthesis of glutathione, leading to the increase in reactive oxygen species (ROS), and compromises the integrity of the intestinal barrier (leaky gut), with a consequent translocation of bacteria and metabolites from the intestinal lumen to extraintestinal sites. Oxidative stress and leaky gut are frequently observed in ASD: these factors contribute to the disruption of various neurological functions through neuroinflammation and disturbances in the neurotransmitters’ biochemical pathways [35,36]. A growing body of evidence suggests that glutamatergic dysfunction, excitotoxicity, and neuroinflammation are intertwined phenomena [37].

The group of ASD children showed a significant increase in Orn plasma levels (+7.6%) compared to the non-ASD group; this increase was again more evident in the NEU subgroup versus the other groups (Table 3). Our findings align with several previous studies reporting varying degrees of increase (from 4% to 30%) in circulating Orn levels in individuals with ASD compared with NT individuals [38,39]. Similar increases have been observed in children with Rett syndrome [40] and in pregnant mothers of autistic newborns [41]. Only a few studies reported decreased or unchanged Orn plasma levels in ASD versus NT controls [21,42]. High Orn blood levels positively correlate with altered psychometric scores in children and adults with ASD, including poor receptive language, reduced socialization, lethargy, stereotypic behaviors, and reduced motor skills [43]. Therefore, we might assume that the increase in concentrations of Orn may contribute to the pathology of ASD. However, it is essential to consider that Orn plasma levels in autistic children and adults may be influenced by several factors, leading to variable results among studies. For example, Clostridium difficile, often associated with gut dysbiosis in autistic individuals, may metabolize Orn via ornithine racemase and D-ornithine 4,5-aminornitase [37].

Asp is involved in multiple metabolic pathways, including urea synthesis, the purine–nucleotide cycle, the malate–aspartate shuttle, and gluconeogenesis [44]. In the brain, it contributes to the production of N-acetylaspartate (NAA) [44]. Despite ASP being a selective agonist for NMDA receptors, there is no agreement on its role in neurotransmission [44]. Our study revealed a significant increase (16.7%) in Asp levels in the ASD group of children compared to the non-ASD group of children, consistent with most results previously published in the literature [8,40,45]. We observed that the increase in Asp plasma level was much more evident in the NEU versus NUT subgroups. At high concentrations, Asp can become a neurotoxin: high levels of Asp can cause Glu accumulation with consequent hyperexcitability and disruption of NMDA receptor signaling [44].

Tau is one of the most abundant AA in various tissues, such as brain, spinal cord, eyes, and leucocytes. Traditionally considered a neurotransmitter with an inhibitory effect, due to its interactions with GABA and Gly receptors, this assumption has been challenged by the absence of Tau in the synaptic vesicles [46]. Tau serves multiple functions in the human body: antioxidant activity, anti-inflammatory effects, osmoregulatory action (a critical brain osmolyte), and involvement in glucose homeostasis. In ASD subjects, Tau plasma levels have been found to be increased [20,22,47], decreased [19,21,33,48], or unchanged [5,7] when compared to NT individuals. This significant heterogeneity in results has been associated with several factors including patient selection, analytical platform used for measuring the AA, severity of the disease, presence of comorbidities, food selectivity, and therapeutic treatment. In our study, Tau levels were found to be twofold higher in the NEU group compared to the NUT, ASD, and non-ASD groups. Elevated Tau levels may represent a compensatory mechanism against ASD pathogenesis, such as oxidative stress [22,47], while low levels may be associated with alterations in brain cell differentiation and in neuronal signaling [49].

The main limitation of this study is the lack of detailed clinical data, which could have enabled a comprehensive characterization of the clinical phenotype. It is important to notice that many covariates—such as clinical factors, neuroimaging findings, genetic variants, and comorbidities—must be accounted for in a sufficiently large sample of children with ASD to fully understand the relationship between metabolic alterations and clinical phenotypes. Changes in certain amino acids (AA) may be linked to higher Autism Diagnostic Observation Schedule (ADOS) scores and more severe DSM-5 ratings, but they could also be influenced by factors like EEG and MRI abnormalities, genetic mutations, psychomotor delays, intellectual disability, language impairment, microbiota imbalances, food selectivity, medication use, and the presence of other comorbidities. Given the heterogeneous nature of these variables in children with ASD, the relationship between AA and clinical outcomes is complex and requires multivariate statistical models for accurate analysis. Clinical studies on ASD are inherently expensive and challenging, both in terms of collecting a broad range of variables and analyzing them with large sample sizes. However, metabolic studies may be more cost-effective if strong preliminary biochemical evidence of altered AA pattern is available. The absence of detailed clinical data in our study limits our ability to draw meaningful conclusions about the links between amino acid changes and various clinical variables, such as the severity of ASD symptoms. While clinical factors such as comorbidities, cognitive function, and behavioral severity could provide valuable insights into the metabolic dysregulations in ASD, our primary objective was to conduct a preliminary pilot study. This cross-sectional survey aimed to confirm or reject the hypothesis that children with ASD exhibit distinct AA patterns compared to non-ASD controls.

Despite these limitations, our findings provide compelling biochemical evidence that alterations in AA profiles may be a characteristic feature of ASD. These results warrant further investigation, particularly through prospective studies that include a broader range of clinical variables. By expanding the scope of future studies to include detailed clinical assessments, we can better understand the potential relationships between metabolic dysregulation and the clinical manifestations of ASD, as well as explore whether specific AA profiles could serve as biomarkers for different ASD subtypes or severity levels.

## 5. Conclusions

In this study, we have highlighted significant alterations in amino acid (AA) profiles in children with ASD, with distinct patterns emerging in two subgroups: the Neurological (NEU) group and the Nutritional (NUT) group. Through multiple comparisons, we identified specific AA alterations associated with neurological impairment in the NEU group, as well as those linked to nutritional dysregulation in the NUT group. The elevated levels of branched-chain amino acids (BCAA), methionine (Met) (likely associated with a B12 deficiency), lysine (Lys), proline (Pro), and alanine (Ala) in the NUT group warrant further investigation as these may indicate underlying metabolic disturbances such as metabolic acidosis. Conversely, the AA alterations observed in the NEU group suggest a potential role for excitotoxicity, oxidative stress, mitochondrial dysfunction, and disturbances in urea cycle metabolism, all of which may contribute to neuronal dysfunction in ASD patients. While our study provides preliminary evidence of these metabolic and neurological mechanisms, further prospective studies with larger cohorts are essential. These studies should aim to explore how specific AA profiles correlate with ASD phenotypes and clinical severity, as well as their potential as biomarkers for personalized therapeutic strategies in ASD.

In conclusion, in this research, by using multiple comparisons, we have emphasized the alterations in AA primarily associated with neurological impairment in the NEU group, as well as those associated with nutritional dysregulation in the NUT group. The elevated levels of BCAA, Met (likely associated to a B12 nutritional deficiency), Lys, Pro, and Ala (possibly indicative of metabolic acidosis) in the NUT group require further investigation. Moreover, the AA alterations characterizing the NEU group also need to be correlated with the distinct mechanism of neurological impairment. There is preliminary evidence on the involvement of excitotoxicity, oxidative stress, impaired mitochondrial function, and imbalance of urea cycle metabolism (as defined in our characterization of AA pattern), and on the interplay of these mechanisms when defining neuronal dysfunction in ASD patients. Further prospective large cohort studies are, however, required to investigate the different AA patterns in relation to ASD phenotypes.

## Figures and Tables

**Table 1:** Comparison of plasma amino acid levels in ASD children versus non-ASD children. Data are expressed as median and interquartile range.

Amino Acid	ASD (n = 1242) (mmol/L)	Non-ASD (n = 488) (mmol/L)	*p*-Value
Essential amino acids			
Histidine	77 (69–85)	75 (67–83)	0.002
Isoleucine	58 (50–69)	55 (47–66)	<0.001
Leucine	117 (102–137)	110 (92–127)	<0.001
Lysine	147 (127–171)	142 (124–167)	0.024
Methionine	21 (18–25)	21 (18–26)	>0.9
Phenylalanine	54 (48–61)	51 (45–57)	<0.001
Threonine	110 (90–131)	107 (89–136)	0.7
Tyrosine	64 (54–76)	62 (52–73)	0.005
Valine	218 (192–253)	205 (168–237)	<0.001
Conditionally essential amino acids			
Arginine	56 (39–73)	59 (43–76)	0.064
Glutamine	462 (413–512)	500 (444–556)	<0.001
Glycine	207 (178–240)	203 (175–232)	0.065
Ornithine	66 (51–87)	61 (49–79)	<0.001
Proline	151 (123–194)	149 (122–181)	0.2
Taurine	78 (61–107)	70 (56–95)	<0.001
Non-essential amino acids			
Alanine	285 (234–360)	271 (225–321)	<0.001
Asparagine	57 (49–67)	57 (49–65)	0.5
Aspartic acid	6.0 (4.0–8.0)	5.0 (4.0–7.0)	<0.001
Citrulline	31 (25–37)	26 (21–31)	<0.001
Glutamic acid	56 (41–78)	53 (37–79)	0.018
Serine	131 (115–150)	128 (112–144)	0.004

**Table 2:** Comparison of amino acid plasma levels in NEU and NUT subgroups with those in non-ASD children. Values are expressed as median and interquartile range.

Amino Acid	NEU (n = 103) (mmol/L)	NUT (n = 78) (mmol/L)	NON-ASD (n = 488) (mmol/L)
Essential amino acids			
Histidine	97 (87–108) ^	87 (79–98) #	75 (67–83)
Isoleucine	71 (62–80) ^	96 (82–114) #	55 (47–66)
Leucine	154 (136–171) ^	177 (160–214) #	110 (92–127)
Lysine	192 (170–220) ^	220 (201–248) #	142 (124–167)
Methionine	24 (21–29) ^	36 (31–42) #	21 (18–26)
Phenylalanine	70 (63–77) ^	74 (67–81) #	51 (45–57)
Threonine	139 (123–159) ^	166 (143–205) #	107 (89–136)
Tyrosine	78 (67–88) ^	110 (88–129) #	62 (52–73)
Valine	249 (224–278) ^	310 (281–383) #	205 (168–237)
Conditionally essential amino acids			
Arginine	30 (17–47) ^	86 (65–106) #	59 (43–76)
Glutamine	427 (378–467) ^	514 (455–568) §	500 (444–556)
Glycine	296 (256–343) ^	221 (196–270) #	203 (175–232)
Ornithine	148 (123–180) ^	90 (75–110) #	61 (49–79)
Proline	197 (161–228) ^	274 (223–349) #	149 (122–181)
Taurine	156 (127–211) ^	83 (62–114) #	70 (56–95)
Non-essential amino acids			
Alanine	376 (327–462) ^	437 (370–497) #	271 (225–321)
Asparagine	74 (66–93) ^	77 (66–88) #	57 (49–65)
Aspartic acid	17 (14–22) ^	7.0 (5.0–9.0) #	5.0 (4.0–7.0)
Citrulline	37 (30–43) ^	31 (24–40) $	26 (21–31)
Glutamic acid	177 (140–214) ^	67 (49–88) £	53 (37–79)
Serine	173 (154–197) ^	161 (143–185) #	128 (112–144)

^ Significant group difference (NEU vs. non-ASD) at q < 0.001 (*p* adjusted after Bonferroni test), # significant group difference (NUT vs. non ASD) at q < 0.001 (*p* adjusted after Bonferroni test), § significant group difference (NUT vs. non ASD) at q < 0.9 (*p* adjusted after Bonferroni test), $ significant group difference (NUT vs. non ASD) at q = 0.003 (*p* adjusted after Bonferroni test), and £ significant group difference (NUT vs. non ASD) at q = 0.033 (*p* adjusted after Bonferroni test).

**Table 3:** Most discriminant amino acids between the subgroups NEU and NUT and the non-ASD and ASD groups.

Amino Acid	NEU vs. non-ASD		NEU vs. ASD	
	Fold Change	%	Fold Change	%
Arginine	0.50	−96.6%	0.53	−86.6%
Ornithine	2.42	+58.8%	2.24	+55.4%
Taurine	2.22	+55.1%	2.00	+50.0%
Aspartic acid	3.40	+70.6%	2.83	+64.7%
Glutamic acid	3.34	+70.1%	3.16	+64.4%
**Amino Acid**	**NUT vs. non-ASD**		**NUT vs. ASD**	
	**Fold Change**	**%**	**Fold Change**	**%**
Lysine	1.55	+35.5%	1.49	+33.2%
Methionine	1.71	+41.7%	1.71	+41.7%
Threonine	1.55	+35.6%	1.51	+33.8%
Tyrosine	1.77	+43.6%	1.72	+41.8%
Isoleucine	1.74	+42.8%	1.65	+39.6%
Leucine	1.60	+37.9%	1.51	+33.9%
Valine	1.51	+33.9%	1.42	+29.7%
Proline	1.83	+45.7%	1.81	+44.9%
Alanine	1.61	+38.0%	1.53	+34.8%

## Data Availability

The datasets generated during and/or analyzed during the current study are not publicly available but are available from the corresponding author on reasonable request.

## Supplementary Materials

The following supporting information can be downloaded at: https://www.mdpi.com/article/10.3390/nu17020274/s1, Supplementary Figure S1. Correlation matrix that shows the relationship between all AA in both ASD group and control group. Spearman correlation coefficient has been computed; Supplementary Figure S2. Screeplot reporting the proportion of variation explained by each principal component performed on ASD group with the complete set of variables. A heuristic “elbow” method has been adopted to determine the number of components retained for subsequent analysis; Supplementary Figure S3. Quality of representation of variables to the PCs obtained on the complete set of variables on ASD group. Supplementary Figure S4. Screeplot reporting the proportion of variation explained by each principal component on analysis performed on Case + Control full case series. Supplementary Figure S5. Quality of representation of variables to the PCs obtained on the complete set of variables on Case + Control full case series. Supplementary Figure S6. Variable correlation plots that show the relationship between all variables on Case + Control full case series.
