# Supplementary material for: Amino Acid Patterns in Children with Autistic Spectrum Disorder: A Preliminary Biochemical Evaluation
Source: Nutrients. 2025 Jan 13;17(2):274. doi: 10.3390/nu17020274 (PMC11767892; doi:10.3390/nu17020274)
Supplement: Supplementary file 1 [file nutrients-17-00274-s001.zip › nutrients-3381573-supplementary.pdf]

Supplementary Figure S1. Correlation matrix that shows the relationship between all AAs in both ASD group and Control group. Spearman correlation coefficient has been computed.

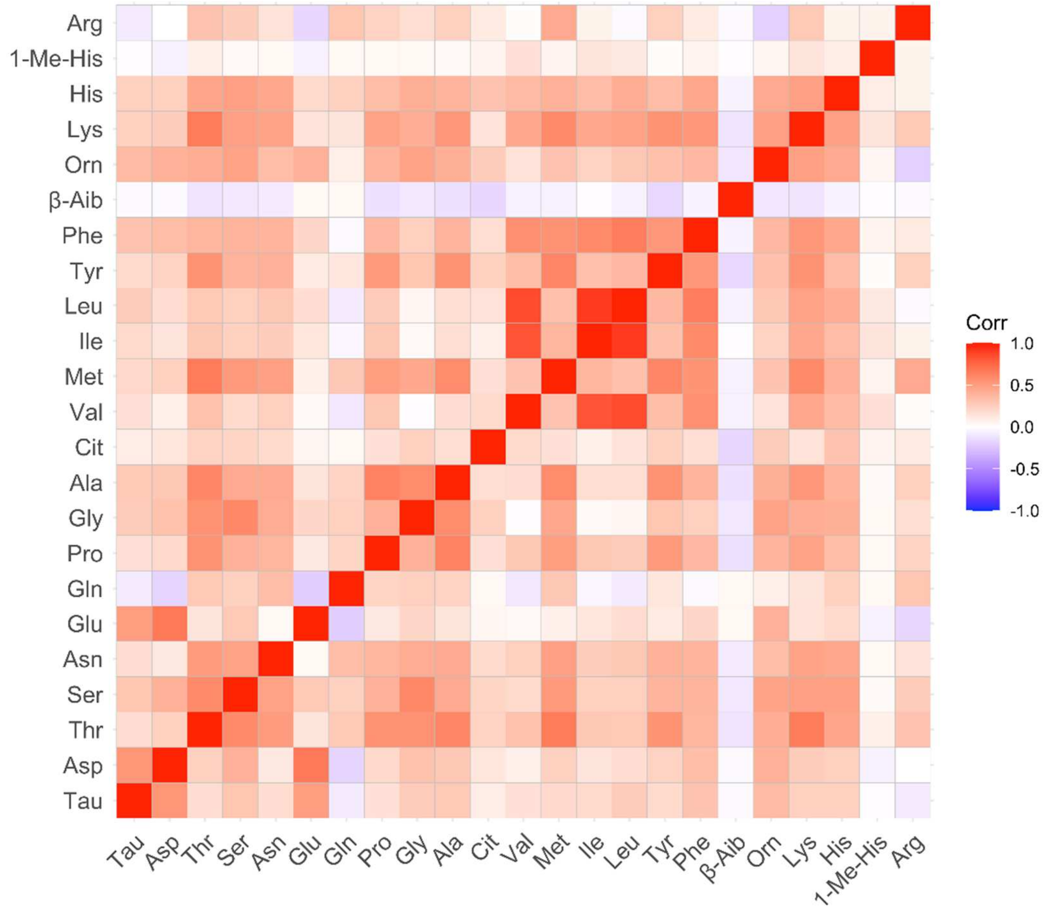

Supplementary Figure S2. Screeplot reporting the proportion of variation explained by each principal component performed on ASD group with the complete set of variables. A heuristic "elbow" method has been adopted to determine the number of components retained for subsequent analysis

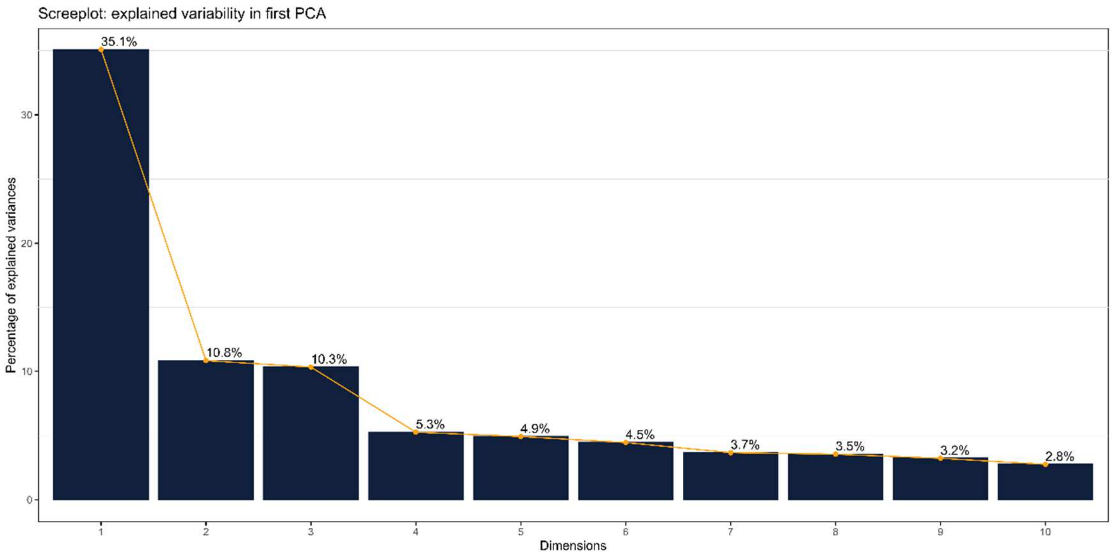

Supplementary Figure S3. Quality of representation of variables to the PCs obtained on the complete set of variables on ASD group.

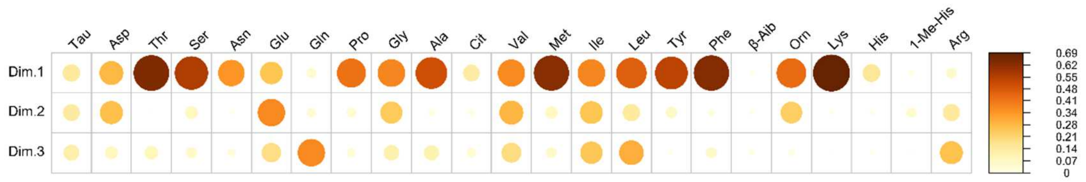

Supplementary Figure S4. Screeplot reporting the proportion of variation explained by each principal component on analysis performed on Case + Control full case series.

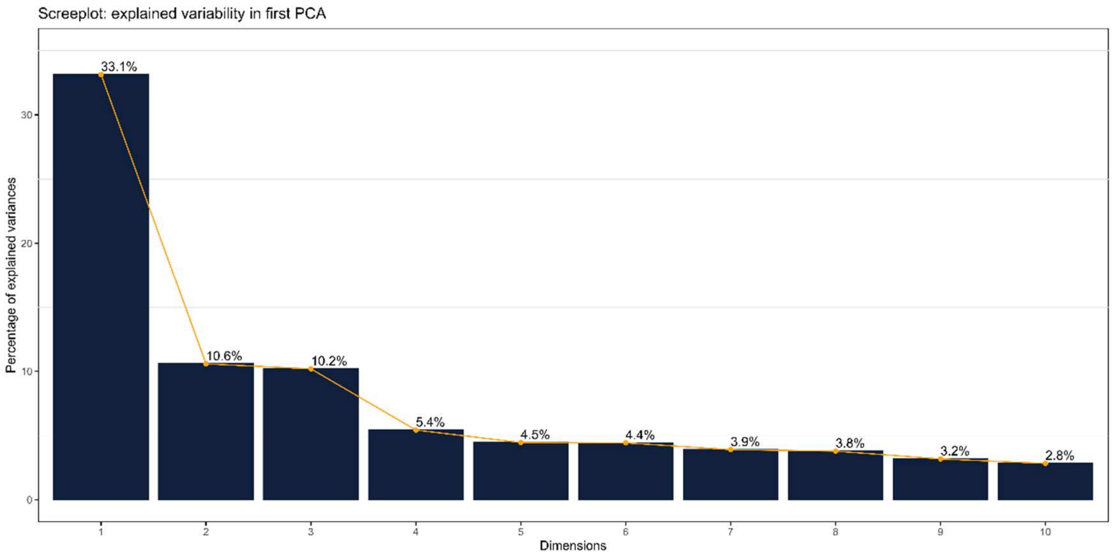

Supplementary Figure S5. Quality of representation of variables to the PCs obtained on the complete set of variables on Case + Control full case series.

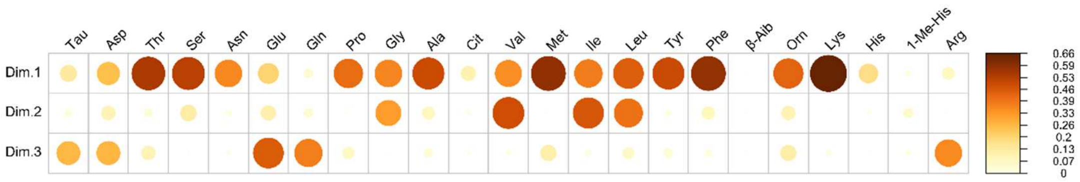

Supplementary Figure S6. Variable correlation plots that show the relationship between all variables on Case + Control full case series.

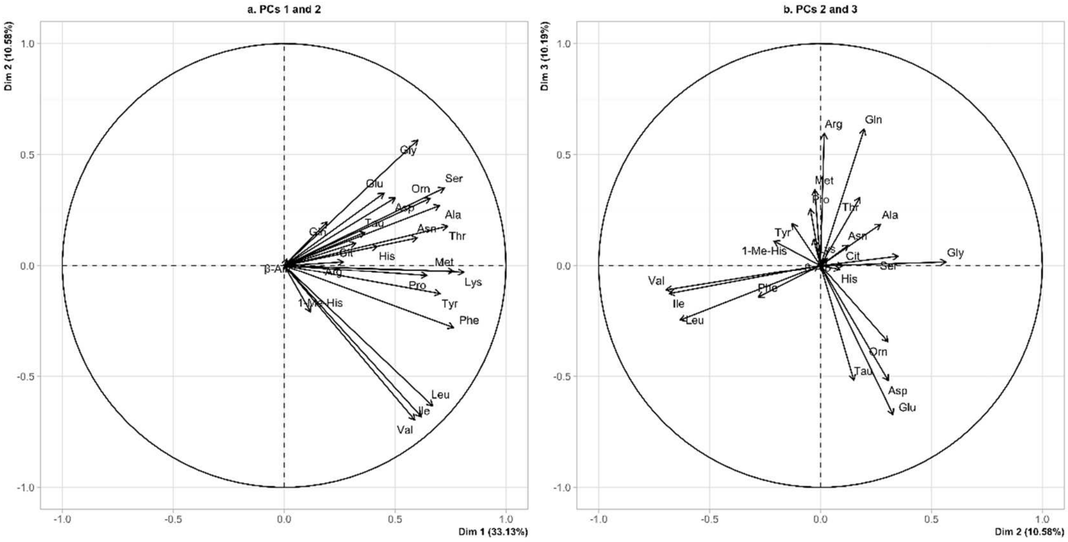

Abbreviations:
"P-Ser", Phosphoserine; "Tau", #Taurine; "P-Etn", #Phosphoethanolamine; "Asp", #Aspartic Acid; "Thr", #Threonine; "Ser", #Serine; "Asn", #Asparagine; "Glu", #Glutamic Acid; "Gln", #Glutamine; "Sar", #Sarcosine; "Aad", # alpha-Aminoadipic Acid; "Hyp", #Hydroxyproline; "Pro", #Proline; "Gly", #Glycine; "Ala", #Alanine, "Cit", #Citrulline; "Abu", #alpha-Aminobutyric Acid; "Val", #Valine; "Cys-Cys", #Cystine; "Met", #Methionine; "Cysta", #Cystathionine; "Ile", #Isoleucine; "Leu", #Leucine; "Tyr", #Tyrosine; "β-Ala", #beta-Alanine; "Phe", #Phenylalanine; "β-Aib", #beta-Aminoisobutyric Acid; "γ-Aib", #gamma-Aminoisobutyric Acid; "Orn", #Ornithine; "Lys", #Lysine; "His", #Histidine; "1-Me-His", #1-Methylhistidine; "3-Me-His", #3-Methylhistidine; "Car", #Carnosine; "Arg", #Arginine; "Hcy" #Homocystine.
